# Product variety and regulation avoidance in the sale of new tobacco products: findings from a point-of-sale survey in Indonesia

**DOI:** 10.1186/s13011-022-00507-w

**Published:** 2022-12-09

**Authors:** Mouhamad Bigwanto, Fathi Muhammad, Sarah Muthia Widad, Laksmana Yudha

**Affiliations:** 1 Doctoral School of Psychology, Eötvös Loránd University, Izabella u. 46, 1064 Budapest, Hungary; 2 Institute of Psychology, Eötvös Loránd University, Izabella u. 46, 1064 Budapest, Hungary; 3 Faculty of Health Sciences, Universitas Muhammadiyah Prof. Dr. HAMKA, Jl. Limau II, Jakarta, 12210 Indonesia; 4 National Committee on Tobacco Control, Jl. Teuku Umar No.8, Jakarta, 10350 Indonesia

**Keywords:** New tobacco products, e-cigarettes, Tobacco control, Point of sale, Indonesia

## Abstract

**Background:**

Since the imposition of the 2018 excise tax, new tobacco products (electronic nicotine delivery system, heated tobacco products, and nicotine pouches) have been considered legal, and their sale has grown rapidly in Indonesia. This study aims to assess the variety of new tobacco products available on the market and evaluate the point-of-sale (POS) environment.

**Methods:**

Data collection was carried out by 12 trained surveyors between March 7 and 13, 2022, in three provinces that had the most e-cigarette users: Yogyakarta, East Kalimantan, and Jakarta. All the vape stores with a Google rating of 4.5 stars or higher and a minimum of 100 reviews were included. In addition to vape stores, the nearest retail stores were included. The data collected included information about available products, product displays, and whether the POS followed tobacco-control measures, such as health warnings and smoke-free regulations.

**Results:**

A total of 27 vape stores and 35 retail stores were observed. The available liquid volumes ranged from 15 to 100 ml, with nicotine levels from 3 to 50 mg. No stores sold flavorless products, and all the products were sold without pictorial health warnings (PHWs). Most of the vape stores (92.6%) reported selling products that used cartoon images or public figures, and most (96.3%) allowed customers to use the products inside the store. Five vape stores and four retail stores reported that they did not require customers to undergo an identity-verification process to buy products. More than half (55.6%) of the vape stores and 46.6% of the retail stores reported that they were located less than 500 m from a school.

**Conclusion:**

The available products were widely varied, and they were easy to access through online and offline stores. The new tobacco products had successfully avoided several tobacco-control measures, including smoke-free regulations and the appearance of PHWs on the products. The government needs to impose regulations sufficient to prevent youth from consuming new tobacco products.

## Background

Over the past decade, the tobacco and nicotine business has changed significantly, especially with the emergence of new tobacco products. Although debates about the long-term health effects of new tobacco products and their effectiveness as a smoking cessation aid are ongoing [1], it has been concluded that all new tobacco products are in fact highly addictive and pose health risks; therefore, these products should not be accessed and used by youth [2].

With at least 16,000 available flavors and various forms of packaging, liquid volumes, nicotine levels, and spare parts [1, 3], e-cigarettes were estimated to have reached USD 15 billion in sales in 2019 [4, 5]. It was reported that heated tobacco products were available in at least 50 countries in 2020 [6]. The three most common new nicotine-based tobacco products available in Indonesia’s market are electronic nicotine delivery systems (ENDS) or e-cigarettes, heated tobacco products (HTPs), and other types of tobacco-processing products such as nicotine pouches [7].

Law No. 7/2021 concerning the Harmonization of Tax Regulations defines ENDS and HTPs as “e-cigarettes” and divides other tobacco products into three categories: molasses, snuff, and chewing tobacco. New tobacco-processing products such as nicotine pouches is categorized as chewing-tobacco products.

Since the imposition of the 2018 excise tax, the Indonesian government has considered new tobacco products legal. The enactment of Law No. 7/2021 concerning the Harmonization of Tax Regulations has strengthened the 2018 decision to impose a tax on these products. The law has categorized ENDS, HTPs, and other new tobacco-processing products as tobacco products.

Despite the new category created by Law No. 7/2021, efforts to subject new tobacco products to tobacco-control measures and regulations have not been realized, including the implementation of pictorial health warnings (PHWs) and smoke-free regulations. Currently, the only existing regulation for the products concerns fiscal policy (excise-tax imposition).

Previous studies of e-cigarettes at the point of sale (POS) have been conducted in Poland, England (London), Guatemala, and New York (Central Harlem). All of these studies focus on marketing activities at the POS [8–11]. To date, no study has assessed the variety of new tobacco products or evaluated the POS situation in Indonesia. Using descriptive qualitative analysis, this study aims to assess the variety of new tobacco products available on the market and to evaluate the POS environment, such as the availability of products, product displays, and whether the POS follows tobacco-control measures.

## Methods

The new tobacco products in this study include Electronic Nicotine Delivery Systems (ENDS), Heated Tobacco Products (HTP), and other tobacco processing products such as nicotine pouches. Only liquids/juice, pods/cartridges, nicotine pouches, and heated tobacco sticks or capsules were included in this study. Devices, spare parts, and accessories associated with the products were excluded.

Before performing field data collection, we carried out an online observation of Tokopedia, the most popular e-commerce site in Indonesia [12]. We searched Tokopedia using six keywords, each of which represented a product type: (1) “vape,” (2) “liquid salt nic,” (3) “pod,” (4) “nicotine pouch,” (5) “Mevius capsule,” and (6) “IQOS HTP.” Due to the lack of search results for the keywords “HTP stick” and “HTP capsule,” we decided that the last two keywords would be more specific if they contained a brand name. From each keyword, we selected the top-five stores and their five best-selling products that meet the inclusion criteria. The collected information included store name, item name, price, and available flavors and volumes.

Field data collection was carried out between March 7 and 13, 2022, in the provinces that had the highest percentages of e-cigarette users according to a recent report from National Basic Health Research: Yogyakarta (7.4%), East Kalimantan (6%), and Jakarta (5.9%) [13]. Data collection at the vape and retail stores was carried out by 12 trained surveyors. All the surveyors were provided with an observation sheet, instructions, and a permit letter from the Ministry of Health.

We selected the vape stores by using the keyword “vape shop” on Google. All the stores with a Google rating of 4.5 stars or higher and a minimum of 100 reviews were included. For retail stores, observations were made at the two largest retail outlets in Indonesia, Indomaret and Alfamart [14]. We selected the retail stores by choosing those closest to the vape stores (i.e., less than 1,000 m away). The distance between the vape and retail shops, on one hand, and schools, on the other, was measured using Google maps and divided into two categories: near schools (less than 500 m away) and far from schools (over 500 m away).

The surveyors interviewed all shopkeepers and input all the answers through an online Google Sheets. The data collected included information about the type of products being sold, the size (liquid volume), the nicotine level, and the most popular brand for each available product type. We also collected information about the cheapest and most expensive items from each available product type.

In addition, we asked the shopkeepers whether they sold any flavorless products or products that use PHWs. We asked them if they sold any products whose packaging features cartoon images or public figures. All the “yes” answers to each question (concerning flavorless products, health warnings, and cartoon or public-figure images) were verified by asking the shopkeeper to display the products and documented by taking pictures of the packaging.

The surveyors also took pictures of the stores’ product displays and observed whether the stores required customers to undergo an age-verification process using an identity card during the transaction and whether any customers used products inside the stores. Due to time constraints, the surveyors observed only two transactions that included this process. Finally, the surveyors identified any promotional materials displayed at the stores.

## Results

### Online stores

Data on 67 products from 17 online stores were collected. E-cigarettes were the most highly available products in the online stores. The price of liquid sold online ranged from IDR 25,000 to IDR 350,000 (Table 1). The liquid volumes available for open-system products were 15 ml, 30 ml, 60 ml, and 100 ml, and those available for closed-system products were 1.5 ml, 1.9 ml, 2 ml, and 2.5 ml. Compared to e-cigarettes, nicotine pouches and HTPs were not widely available in the online stores.

**Table 1:** Result from Online Stores on Tokopedia

Keywords	Products (Store)	Price (IDR)
Vape	22 (5 stores)	51,000-163,000
Liquid Salt Nic	22 (5 stores)	25,000-210,000
Pod	15 (3 stores)	64,900 − 350,000
Nicotine Pouch	1 (1 store)	30,000
Capsul Mevius	1 (1 store)	265,000
HTP IQOS	6 (2 store)	34,000-352,110

### Vape stores

A total of 27 vape stores met the criteria: 12 from Yogyakarta, 10 from Jakarta, and 5 from East Kalimantan (Samarinda). All the stores reported selling e-cigarette products for both open and closed systems. The volumes of liquids were the same as those in online stores, with nicotine levels ranging from 3 mg, 6 mg, and 9 mg to 50 mg. One shop sold 50-ml liquids. For closed-system products, the available packaging sizes ranged from one to three pods.

**Fig. 1:**
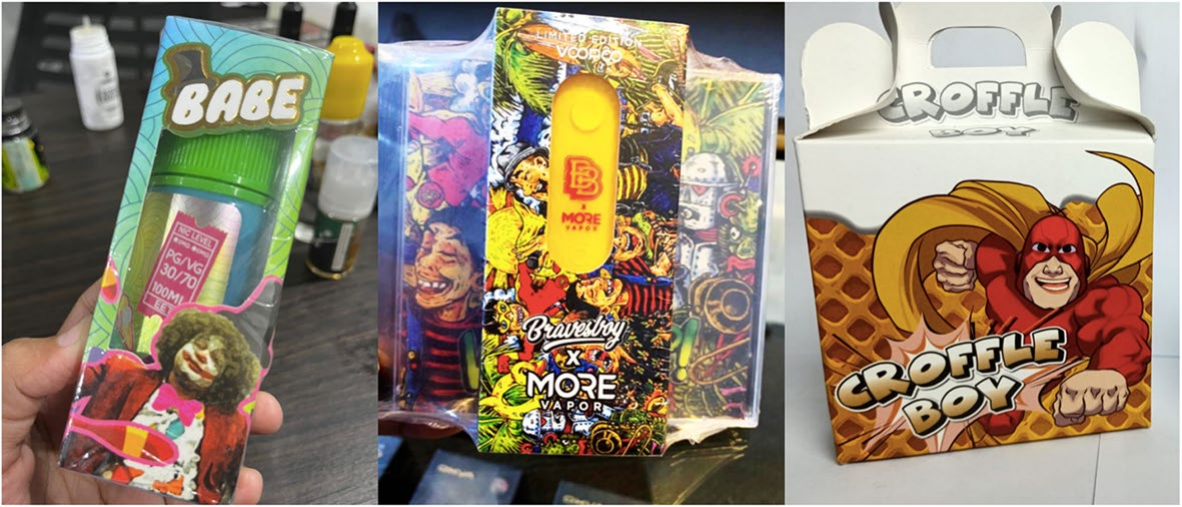
ENDS Packaging Contain Images of Comedian, Musicians (Public Figures), and Cartoon

No vape shop sold flavorless products. All the products were sold without PHWs. Most of the vape stores (92.6%) reported selling products with packaging that featured cartoon images or images of public figures (Fig. 1). Six vape stores reportedly did not sell vape products exclusively; they sold other products as well, including ice cream, fashion accessories, clothes, and bags. At least two stores reported providing coffeeshop services in addition to selling vape products. Moreover, 15 of the 27 vape stores were located less than 500 m from a school.

**Fig. 2:**
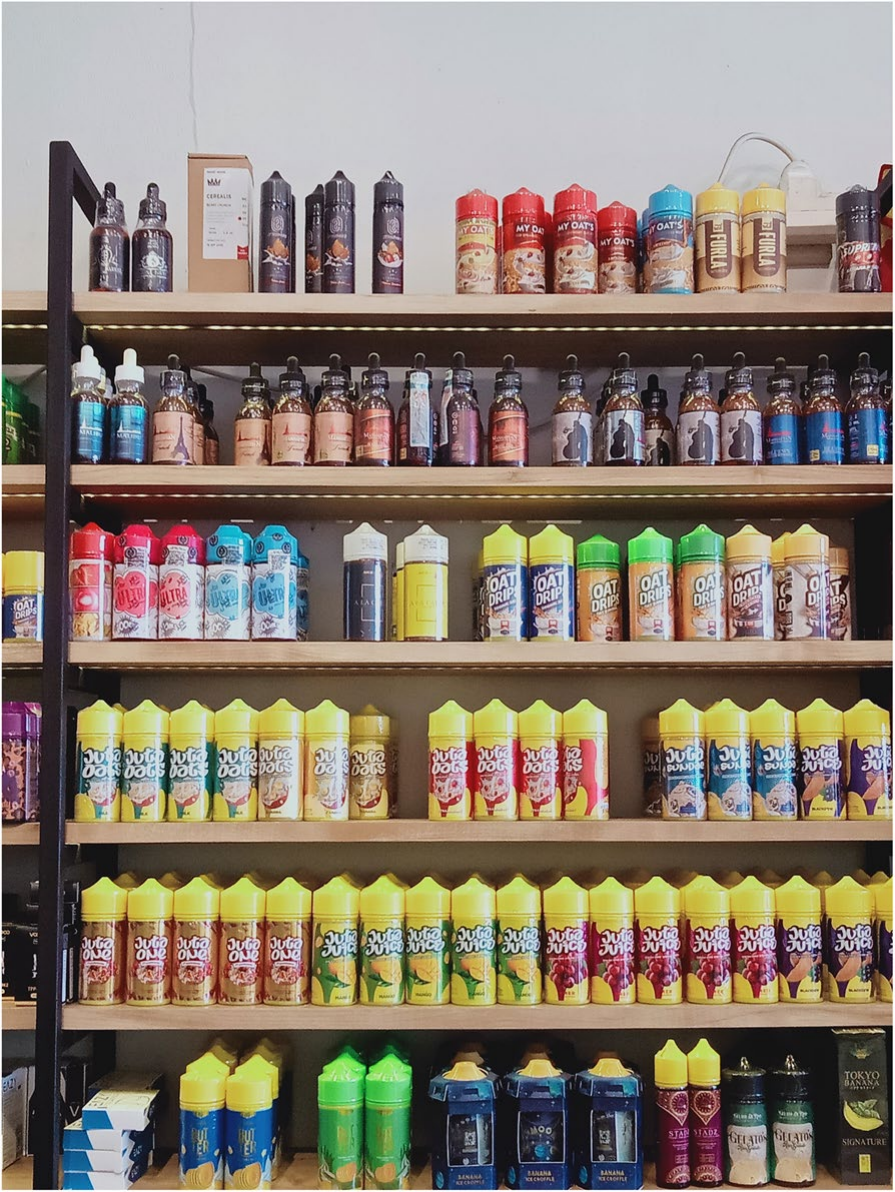
Products Display at Vape Store

The majority of the stores (96.3%) allowed their customers to use products inside the store. Five stores reported that they did not require customers to undergo an identity-verification process to buy products. At least 10 stores provided product-promotion materials such as pamphlets, catalogs, and magazines. The cheapest price reported was IDR 25,000 for liquids and IDR 40,000 for pods. The stores’ product displays resembled mini-billboards (Fig. 2), and one shop sold products on a self-service basis.

### Retail stores

**Fig. 3:**
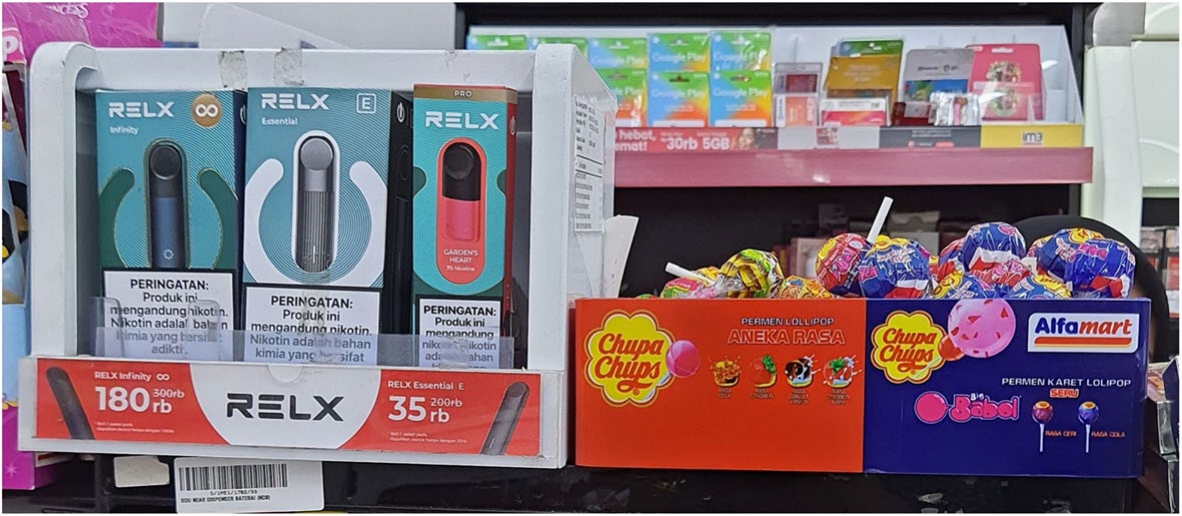
ENDS Products Placed next to Candy at Retail Store

A total of 35 retail stores were observed, and 13 stores (37%) reported selling e-cigarette (closed-system) products. Nine stores sold liquid and nicotine pouches (20 bags per pack for IDR 30,000) in addition to closed-system products. No retail store sold flavorless products, and all the products were sold without PHWs. Four of the 13 stores reported not verifying the identity of customers who wanted to buy tobacco products. New tobacco products were displayed in the same way as cigarette products; some products were placed in front of the cashier as well as alongside products such as candy and chocolate (Fig. 3). In addition, six of the 13 retail stores that sold new tobacco products were located less than 500 m from a school.

## Discussion

Compared to e-cigarettes and nicotine pouches, HTPs (including those in capsule form) were more difficult to find in stores. However, these products were available and could be purchased in online stores. E-cigarettes and nicotine pouches were also more accessible than heated tobacco products. These products were available in retail stores, and some could be purchased without an identity-verification process. This is very concerning, because over half (56%) of the vape stores and 46% of the retail stores were located under 500 m from a school, and the retail stores displayed these products in such a way that children could easily see them. Some retail stores even displayed e-cigarette products in front of the cashier or next to products favored by children, such as chocolate or candy.

It was previously reported that the prevalence of e-cigarette use in Indonesia among adolescents aged 10–18 years had increased rapidly, from 1.2% to 2016 to 10.9% in 2018 [13, 15]. A local study among high school students in Jakarta in 2019 revealed that 11.8% of the students were e-cigarette users and more than half (51.1%) were dual users [16]. This trend will probably continue, because the products have been promoted freely and without restrictions in Indonesia. A systematic review of epidemiological and laboratory studies revealed that using e-cigarettes can lead to asthma and chronic obstructive pulmonary disease in both the adolescent and adult population [17].

All the stores reported that they sold only flavored nicotine products. This finding strengthens the validity of previous results concerning the importance of the flavor component in making e-cigarettes more attractive to youth [18–20]. Therefore, limiting or banning flavor could be the most significant means of preventing youth from adopting the use of e-cigarettes [21, 22]. Several studies have shown that the flavors in e-cigarettes can cause health problems [23, 24].

Various product sizes and forms of packaging were available on the market. Some of them were shaped like food snacks. Of the vape stores, 93% reported selling products with packaging that used cartoon images or images of public figures. Such images make the product’s appearance more attractive to youth. Although the Ministry of Finance issued Regulation No. 193/PMK.010/21, which limits liquid volumes to 15 ml, 30 ml, and 60 ml, in 2022, this provision does not regulate the form of packaging. To date, no government body has set packaging standards for new tobacco products.

Since the enactment of Law No. 7/2021 concerning the Harmonization of Tax Regulations, which categorizes e-cigarettes and other tobacco-processing products as tobacco products, the Ministry of Health has had an opportunity to implement PHWs in new tobacco products. It was previously reported that the use of PHWs on cigarette packs was considered effective enough to increase awareness of the dangers of cigarettes, especially among early adopters [25].

The product displays at the vape stores were much more varied than those at the retail stores. One vape shop allowed customers to shop on a self-service basis. Some of the vape stores paired new tobacco products with other consumer goods or services, such as ice cream and coffeeshop services. These findings may indicate that vaping is increasingly viewed as normal and a recreational rather than a smoking-cessation tool. A previous study found that smoking cessation was a less important motivation than a recreational for youth who used e-cigarettes [20]. Most of the vape stores (96%) allowed their customers to use electronic products inside the store. This result is in line with a 2017 US study in which vapers reported unrestricted use of e-cigarettes in public places, including indoor areas [26].

Due to the large of products variety (nicotine level, forms, etc.), size (liquid volume), and limited studies on the affordability of new tobacco products in Indonesia, it is difficult to determine whether the products are affordable or not. Future studies could investigate the affordability of new tobacco products in relation to cigarette prices. However, it is quite difficult to compare these two products due to the different units of measure as well as the wide variety of nicotine levels, especially in e-cigarettes.

### Study limitations

This study was conducted in only three provinces, and there was one province (East Kalimantan) where data collection was conducted only in the capital city. In addition, the information collected for this study excluded devices, spare parts, and accessories associated with the products.

## Conclusion

The new tobacco products were widely available, easy to access, and successfully avoided tobacco control measures. Besides being offered in a variety of flavors, the packaging of new tobacco products was designed to be very attractive. The government needs to provide adequate regulation to prevent youth from consuming the products, this includes the implementation of pictorial health warnings, and applying standards packaging (size and form). Moreover, since the products displayed resemble mini billboards and most stores allow the use of the products inside the stores, the local governments need to include new tobacco products under their smoke-free regulations.

## Data Availability

The datasets used and/or analyzed during the current study are available from the corresponding author on reasonable request.

## Abbreviations

HTP: Heated tobacco product
ENDS: Electronic nicotine delivery system
POS: Point of sale
PHW: Pictorial health warning
IDR: Indonesian Rupiah
USD: United States dollar
